# The Relationship Between Prior Cancer Diagnosis and All-Cause Dementia Progression Among U.S. Adults

**DOI:** 10.1093/geroni/igab046.383

**Published:** 2021-12-17

**Authors:** Mackenzie Fowler, Nicole Wright, Kristen Triebel, Gabrielle Rocque, Ryan Irvin, Richard Kennedy

**Affiliations:** University of Alabama at Birmingham, Birmingham, Alabama, United States

## Abstract

Cancer-related cognitive impairment is a common effect of cancer that shares symptoms with dementia. Only one study examined cancer’s longitudinal association with dementia. This analysis expands to a larger clinical sample. Electronic health record data were extracted from July 2003-February 2020. Baseline cognition/progression on the Alabama Brief Cognitive Screener (ABCs) by cancer history were assessed using linear mixed effects models, with interaction by race. After adjustment for demographics/socioeconomics, those with cancer history had higher baseline cognition (□: 1.49 [0.91-2.07]), and declined slower (□: 0.40 [0.08-0.71]) than those without. Health behaviors/comorbidities attenuated this association. Non-Hispanic Blacks with cancer history demonstrated lower cognition throughout follow-up compared to non-Hispanic Whites / other race/ethnicities with cancer history and participants without cancer history. Health behaviors/comorbidities confound and race modifies the relationship between cancer and dementia. Exploring the role of health behaviors/comorbidities on this association and causes of racial disparities is needed.

